# Leishmaniosis caused by *Leishmania infantum* in ferrets: Update review

**DOI:** 10.1016/j.vas.2021.100229

**Published:** 2021-12-27

**Authors:** Sergio Villanueva-Saz, Jacobo Giner, Diana Marteles, Maite Verde, Andrés Yzuel, Cristina Riera, Roser Fisa, Magdalena Alcover, Antonio Fernández

**Affiliations:** a Department of Animal Pathology, Veterinary Faculty, University of Zaragoza, Spain; b Clinical Immunology Laboratory, Veterinary Faculty, University of Zaragoza, Spain; c Instituto Agroalimentario de Aragón-IA2 (Universidad de Zaragoza-CITA), Spain; d Menescalia Veterinary Clinic, Ismael Merlo Actor, 5, 46020 Valencia, Spain; e Departament de Biologia, Salut i Medi Ambient, Facultat de Farmacia, Universitat de Barcelona, Spain

**Keywords:** Diagnosis, Ferret, Leishmania infantum, Mustela putorius furo, Prevention, Treatment

## Abstract

Leishmaniosis in domestic ferrets (*Mustela putorius furo*) is a disease caused by *Leishmania infantum*, a parasite transmitted through the bite of an infected female phlebotomine sand fly. Among vertebrates, the dog is the primary domestic reservoir of the parasite; however, other domestic animals can be implicated such as cats. The first description of a clinical case of leishmaniosis in domestic ferrets was reported recently. As a result, new knowledge has been published including empirically based treatment protocols, confirmatory techniques to detect the presence of the parasite infection and seasonal variation in the antibodies against *Leishmania* in apparently healthy domestic ferrets. The most common clinical signs observed are enlargement of peripheral lymph nodes and skin lesions such as papular and/or ulcerative dermatitis. Additionally, the most frequent laboratory alterations seen are hyperproteinaemia with hyperglobulinaemia and biochemical analytes alterations depending on the affected tissue. Two different therapeutic protocols have been described to treat domestic ferrets with leishmaniosis: meglumine antimoniate plus allopurinol protocol or miltefosine plus allopurinol protocol. These treatment protocols seemed to be able to control the *Leishmania* infection, although the presence of xanthinuria could be detected. The susceptibility of domestic ferrets to *Leishmania infantum*, the clinical picture, treatment of infected animals and prevention are poorly understood, due to the scarcity of recent description in the literature. Different proposed diagnostic algorithms have been included for domestic ferrets with suspected leishmaniosis, clinically healthy domestic ferrets and animals as blood donors. In this sense, the present review provides updated data on scientific knowledge of leishmaniosis in ferrets.

## Graphical Abstract

**Figure:**
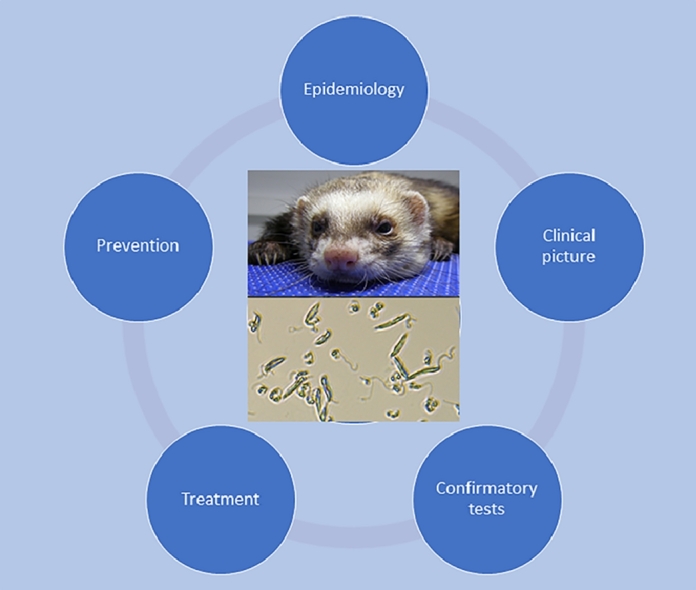

## 1 Introduction

Leishmaniosis is a zoonotic infection caused by *Leishmania infantum*, which mainly occurs with chronic clinical forms, and transmitted through the bite of infected females phlebotomine sand flies in Southern Europe. Dogs are considered the primary domestic reservoir of the parasite causing human visceral leishmaniasis. However, other animals including cats can be implicated as additional reservoir.

In Tunisia, the first report of *Leishmania* infection in a dog was described in 1908 (Nicolle & Comte, 1908), whilst the first report of feline leishmaniosis (FeL) was described in 1912 in Algeria (Sergent, Lonbard, & Quilichini, 1912). Since then, the scientific information about *L. infantum* infection focused on dogs, and more recently in cats, has increased. One-hundred-eight years after the first record of FeL, the first clinical case of leishmaniosis in a domestic ferret (Giner et al., 2020a) was published in Spain. However, the susceptibility of domestic ferrets (*Mustela putorius furo*) to *L. infantum*, the clinical pictures, management and treatment of infected animals are poorly understood, associated to the recent description in the literature.

## 2 Material and methods

### 2.1 Search and eligibility criteria

A bibliographic search was carried out in the database of Pubmed. The following combination of keywords was used/cross referenced: Leish* AND (control OR disease OR diagnosis OR epidem* OR infection OR one health OR reservoir OR transmission OR treatment) AND (ferret). Other inclusion criteria were the language (English) and date of publication (between January 1, 1990 and September 31, 2021). This review was carried out essentially based on guidelines outilined in the study published in Research Synthesis Methods (Polanin, Piggot, Espelage, & Grotpeter, 2019).

## 3 Results and discussion

A total of 4 articles were included in this review. The number of domestic ferrets in all reported studies was 23. The most relevant information obtained is presented based on the following topics including epidemiology, clinical manifestations and laboratory abnormalities, diagnosis, treatment and prevention.

### 3.1 Epidemiology

There is a lack of clinical description of the disease and epidemiological information of the infection in terms of seroprevalence and prevalence rates. As in cats, the prevalence of infection in endemic areas should be considered lower compared to dogs, due to the lack of infection surveys and clinical descriptions of the disease. Cases of FeL have been reported and described in several countries in Europe, South America, the US and Asia (Pereira & Maia, 2021). These cases have been described from traditionally endemic areas of canine leishmaniosis (CanL) where sand fly transmission can occur during most parts of the year, and the main mode of transmission route is through the bite of the infected female sand fly to human or animals.

Other non-vectorial transmission routes have not been described; however, it is possible to detect the presence of *Leishmania* spp. DNA in peripheral blood samples obtained from a clinically affected domestic ferret (Giner et al., 2020a), transfusion-transmitted leishmaniosis should be taken account in this species as well as it can occur in humans and dogs.

Leishmaniosis in domestic ferrets could be underdiagnosed because of lack of specific commercially available confirmatory techniques for domestic ferrets to detect the infection and the scarce clinical description of the disease. Immune response could be playing an important role in terms of susceptibility/resistance to *L. infantum* infection. In domestic ferrets with leishmaniosis, the presence of concurrent diseases was reported in a clinical case associated with impaired immune response (Giner et al., 2020b), whilst a second clinical case was not associated with concurrent immunosuppressive conditions (Giner et al., 2021a). In this regard, it is possible that the immune response elicited against the parasite in domestic ferrets was similar to that of dogs (Ordeix, Montserrat-Sangrà, Martinez-Orellana, Baxarias, & Solano-Gallego, 2019) and cats with *L. infantum* specific IFN-γ production (Priolo et al., 2019)

Although dogs are the most important hosts of the parasite, domestic ferrets could be among the potential domestic reservoirs for *L. infantum*. However, conducting research is necessary for the evaluation of the infectiveness of domestic ferret with leishmaniosis to sand flies based on xenodiagnosis. Further studies are necessary to elucidate their epidemiological roles (Giner et al., 2020a, 2021a).

### 3.2 Clinical manifestations and laboratory abnormalities

Domestic ferret leishmaniosis is a multiorgan disease that affects different organs and tissues with the presence of non-specific clinicopathological abnormalities detected and clinical signs observed in two clinical cases at the moment. The more relevant clinical manifestation found in these domestic ferrets is skin lesions which could be detected along with other laboratory abnormalities (Giner et al., 2020a, 2021a). One domestic ferret presented a nonpruritic erythematous and an edematous papular painless skin lesion on the right ear pinna compatible with papular dermatitis or nodular lesion associated to the inoculation site. The other domestic ferret presented a nonpruritic ulceration of the lower lip. In both cases, a severe chronic diffuse pyogranulomatous dermatitis was detected by histological examination. In this sense, leishmaniosis could induce in domestic ferrets a pyogranulomatous and granulomatous inflammation, being necessary to rule out other causes.

In the first clinical case reported, no other apparent clinical signs were detected. However, an immunosuppressive status and elevation of the parasitic load was related to immunosuppressive therapy. Furthermore, *Leishmania* infection probably could exacerbate the immunosuppression status of the patient because cryptosporidiosis with intestinal and pulmonary signs was detected several months after *Leishmania* infection had been confirmed (Giner et al., 2020b). In the second clinical case, other clinical signs detected were peripheral lymphadenomegaly and splenomegaly.

There is a published case report about clinical leishmaniosis in another animal belonging to the same carnivorous mammals’ family, Mustelidae, a captive Eurasian otter (*Lutra lutra*) with epistaxis and non-specific clinical signs such as anorexia, apathy, and weight loss (Cantos-Barreda et al., 2020).

Hyperglobulinemia was the most common laboratory abnormality detected in both domestic ferrets as well as in the captive Eurasian otter leishmaniosis clinical case. Clinical leishmaniosis should be considered in the differential diagnosis of hyperglobulinemia in domestic ferrets, a clinicopathological abnormality usually associated with a variety of infections in this species including systemic mycoses, viruses and finally certain neoplasia (Melillo, 2013). Serum protein electrophoresis is a crucial biochemical technique used for the investigation of a normal distribution of serum protein fractions (albumin, *α*−1, *α*−2, *β*−1, *β*−2 and *γ*). In small animal veterinary medicine, different serum protein electrophoresis patterns could be detected, from normal pattern to acute-phase protein responses, polyclonal gammopathies, oligoclonal gammopathies or also called restricted polyclonal gammopathies and finally monoclonal or paraproteinemias. In canine leishmaniosis, three different gammopathies patterns are associated to the disease including polyclonal, oligoclonal, biclonal or monoclonal gammpathy, being the polyclonal pattern, the most common gammopathy detected (Paltrinieri, Gradoni, Roura, Zatelli, & Zini, 2016).

Furthermore, another laboratory finding detected in one of the patients was a high serum enzymes activity including alanine aminotransferase, alkaline phosphatase and gamma-glutamyl transferase. The cause of the elevation of these liver parameters and whether these were related to leishmaniosis as occurs occasionally in dogs (Villanueva- Saz, Peréz, Yzuel, Fernández, & Verde, 2020) cannot be determined because liver biopsies were not obtained in the domestic ferret. Bile culture and abdominal ultrasound were performed in the domestic ferret of this report with negative results; however, the favourable response to anti-*Leishmania* treatment could be considered as indirect indicator that *Leishmania* parasites might have some role in the pathogenesis of the hepatic disease.

### 3.3 Diagnosis

**Fig. 1:**
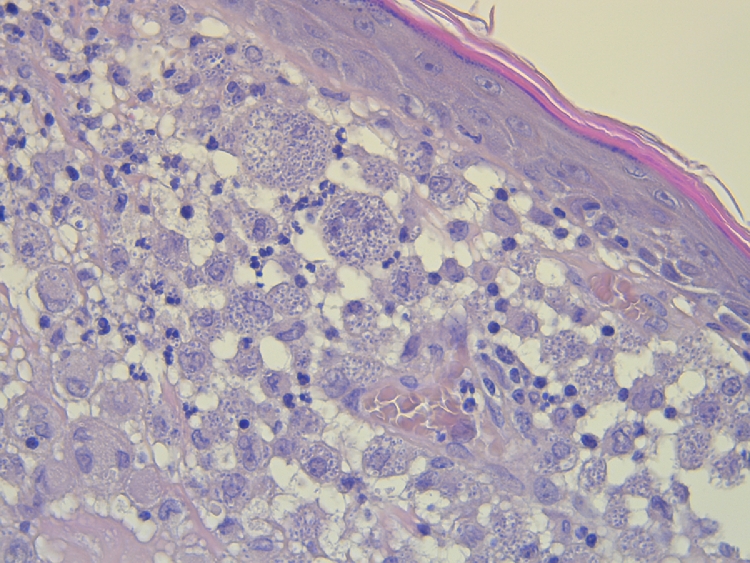
Histological section of skin from a domestic ferret with suspected of leishmaniosis. Inflammatory lesion reveals the presence of macrophages and multinucleate giant cells. The cytoplasm of these cells is laden with *Leishmania* spp. amastigotes. Hematoxylin and eosin stain (x40 objective).

**Fig. 2:**
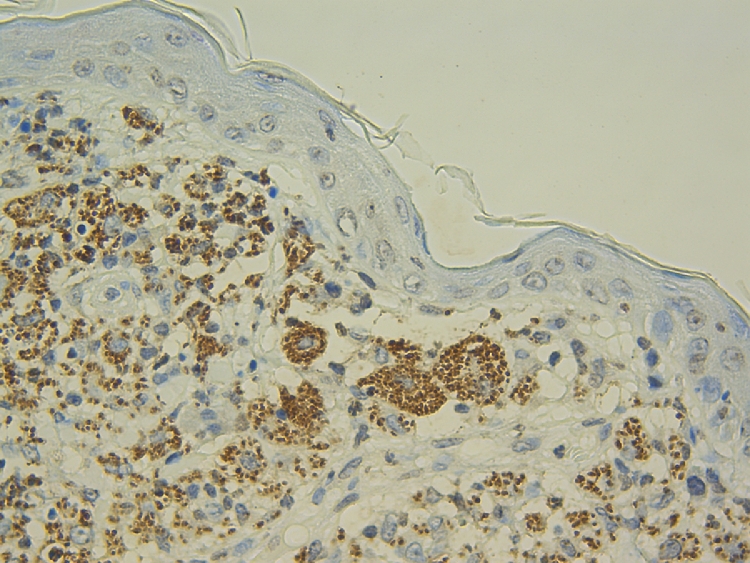
Immunohistochemical staining labeling of *Leishmania* spp. amastigotes (skin of a domestic ferret with suspected of leishmaniosis). The amastigotes parasites labelled in brown (x40 objective).

The same confirmatory techniques to detect the presence of *L. infantum* infection in dogs and cats can be available for domestic ferrets. Parasitological methods include all confirmatory techniques with direct observation of the parasite such as cytology, where amastigotes can be found in infected macrophages. Cytological study from lymph nodes samples in case of lymphadenomegaly can confirm the presence of the *Leishmania* parasite. Other tissues such as bone marrow are not always accessible in domestic ferrets due to the small size of the patient. Histology (Fig. 1) and histopathological evaluation are recommended in cases where cytology of the lesions is classified as a negative result for the presence of *Leishmania* amastigotes. The presence of granulomatous inflammation pattern in absence of intracellular *Leishmania* amastigotes should be evaluated considering specific immunohistochemistry using a hyperimmune serum obtained by experimental animal immunized with *L. infantum* antigen due to the lack of commercial specific primary antibodies to be used in the immunohistochemical diagnosis of leishmaniosis (Fig. 2).

**Fig. 3:**
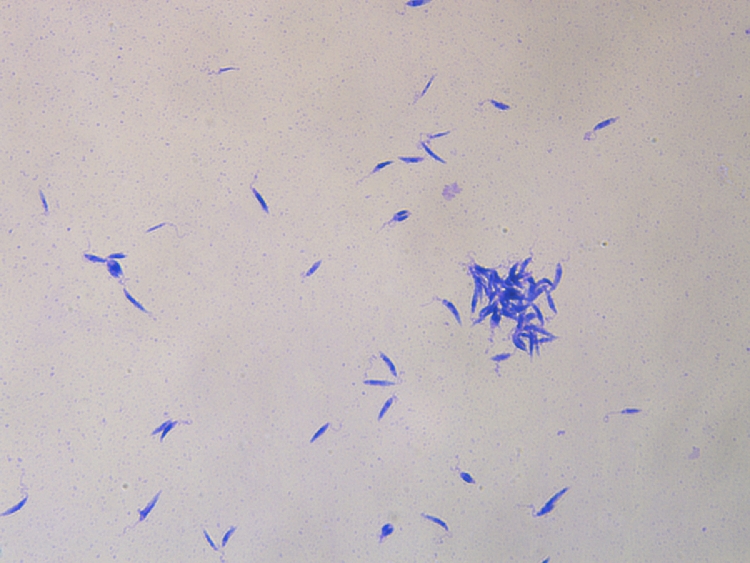
*Leishmania* spp. promastigotes isolated by NNN culture obtained from a domestic ferret with suspected of leishmaniosis. Giemsa stain (x40 objective).

Culture and *Leishmania* isolation are another parasitological method that can be used and different types of media can be employed, being the most common the biphasic Novy-McNeal-Nicolle medium (NNN) and the Schneider´s medium. The NNN is a culture medium prepared preferably with rabbit blood and agar, although other blood from different animals can be used, whilst the liquid phase contains fetal calf serum, antibiotics and other supplementary components necessary to produce a great number of promastigotes. By contrast, Schneider´s medium is a monophasic liquid medium prepared with different components, the most important medium being Schneider′s insect medium. (Castelli et al., 2014). This type of diagnostic procedure requires special laboratories including trained personnel and class II biosafety cabinets, and two main disadvantages: the possibility of bacterial contamination of the medium and the time consuming for *Leishmania* species to be able to grow. Nevertheless, the isolation and subsequent positive *Leishmania* culture allows to establish the cause-effect relationship in the animal with leishmaniosis (Fig. 3).

Serology is a methodology based on the detection of specific anti-IgG antibodies against *L. infantum* using different techniques including ELISA, immunofluorescence antibody test (IFAT) and western blot (WB). When the disease is described for the first time in other species, it is necessary to establish the cut off setting. This cut off could be similar or different to other species such as dogs and cats. In domestic ferrets (Giner, 2020a) and cats (Iatta et al., 2020), cut off was set at 1:80 dilution for IFAT, whilst, in dogs was set from 1/20 to 1/160 (Santarém et al., 2020). In general, serological techniques must be adapted to the animal species, first, for example, using different FITC-conjugate for IFAT and then validated to evaluate diagnostic measures including sensitivity and specificity.

Among serological tests to investigate *Leishmania* infection in cats, WB technique has the best diagnostic performance in terms of sensitivity (Alcover et al., 2021a; Persichetti et al., 2017). In cats infected with *L. infantum*, WB can detect different antigen bands including 14 kDa, 16 kDa, 18 kDa, 20 kDa, 24 kDa, 36 kDa and 46 kDa, the most frequent positive bands detected were 46 kDa and 16 kDa (Alcover, Riera, & Fisa, 2021). In particular, WB in domestic ferrets with clinical leishmaniosis presented reactivity against the 14 and/or 16 kDa bands as described previously in cats (Alcover et al., 2021a; Giner et al., 2020a).

Recent evidence suggests that a variation in the anti-*Leishmania* antibodies can be detected during the sand fly transmission period and the following non sand fly transmission period in apparently healthy domestic ferrets in natural conditions (Villanueva-Saz et al., 2021). This fact is important from a point of sampling time due to the presence of apparently healthy seropositive domestic ferrets during the transmission period, making it necessary to reassess the serological status during the non-transmission period to avoid unnecessary treatment based on antibody titers obtained during transmission period (Villanueva-Saz et al., 2021). Other factors that should be taken into account is the fact that serological methods should be validated to be used in domestic ferrets. For this purpose, two different steps should be carried out including a first step based on design, adaptation an preparation of the techniques and the second step with a field validation on a domestic ferret population, possibly with two subpopulations from endemic and non-endemic areas. Finally, serological methods for use in cats or dogs would give a negative result in seropositive domestic ferrets.

The presence of *Leishmania* spp. DNA in different types of matrix (EDTA-blood, paraffin-embedded tissue specimens, Whatman filter paper, bone marrow and lymph node fine-needle aspiration, among others) could be evaluated by real time PCR to determine the parasitic load (Giner et al., 2020a). For a better diagnostic management, a combination of confirmatory techniques with different nature, including a qPCR technique and a quantitative serology, should be performed.

**Fig. 4:**
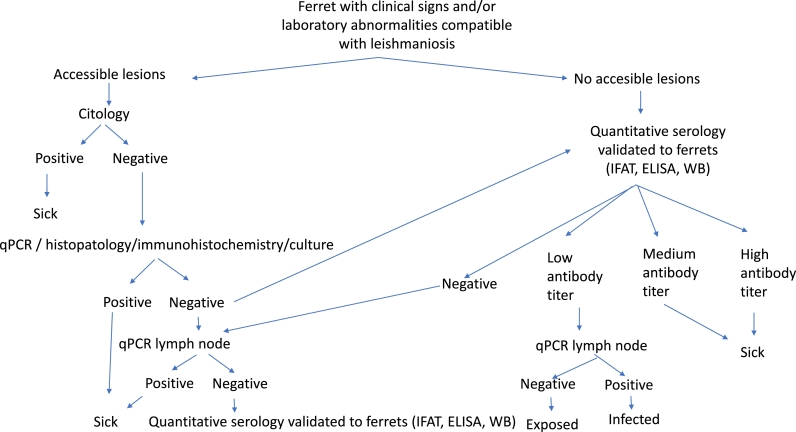
Diagnostic algorithm for domestic ferrets with suspected of leishmaniosis.

In conclusion, the diagnosis of the disease in domestic ferrets is similar to dogs and cats. It requires the integration of the clinical picture and the laboratory abnormalities detected in the laboratory tests performed (complete blood count, complete biochemical profile, urinalysis and serum protein electrophoresis) together with a positive result of any of the confirmatory techniques described previously. In this sense, the diagnostic algorithm for domestic ferrets with suspected leishmaniosis is included (Fig. 4).

### 3.5 Treatment

The information on the treatment of domestic ferrets with leishmaniosis is extremely scarce because only two reports have been published. Two different therapeutic protocols have been described to treat domestic ferret with leishmaniosis: meglumine antimoniate plus allopurinol (Giner et al., 2020b) or miltefosine plus allopurinol (Giner et al., 2021a). Although combined therapeutic protocols based on these drugs were well tolerated in those patients, information is lacking on pharmacological characteristics of these drugs in domestic ferrets. In addition, in the captive Eurasian otter clinical case, allopurinol alone was administrated as treatment protocol (Cantos-Barreda et al., 2020).

Allopurinol is a compound that blocks RNA synthesis in *Leishmania*. Consequently, a negative effect in parasite multiplication is produced. The length of allopurinol treatment in dogs and cats depends on the response to treatment and the individual tolerance to this drug (Pennisi et al., 2015; Solano-Gallego et al., 2011). Although it is well tolerated by dogs, different side effects are associated with its administration including xanthinuria, and the occurrence of itching specifically in some dogs with continuous long-term allopurinol therapy (Manna et al., 2014; Torres et al., 2016). In dogs, xanthinuria can develop during the first few weeks after starting allopurinol treatment (Torres et al., 2016). Increased levels of xanthine could produce more severe situations such as xanthine urolithiasis and renal mineralization. The presence of xanthine crystals is a result of the inhibition of the xanthine oxidase enzyme, which is part of the pathway to allopurinol degradation. Usually, xanthine crystalluria is identified with the morphology of crystals without the need of evaluation by optical crystallography, although sometimes these crystals are impossible to distinguish from others (ammonium biurate and amorphous urate crystals) with similar appearance by light microscopy. In domestic ferrets, the presence of xanthinuria could be detected associated to the allopurinol treatment in some animals, whilst, other domestic ferrets did not develop this type of urine crystal. For thus, domestic ferrets receiving therapy should be monitored for the development of urinary adverse effects from the beginning of treatment and urinalysis should be systematically used in follow-up.

Meglumine antimoniate is one of the first-choice drug for the treatment of CanL (Oliva et al., 2010). This compound causes a marked decrease in the parasitic load in dogs during the treatment (Manna et al., 2014). The main side effects in dogs are potential nephrotoxicity and cutaneous abscesses/cellulitis (Solano-Gallego et al., 2011).

**Fig. 5:**
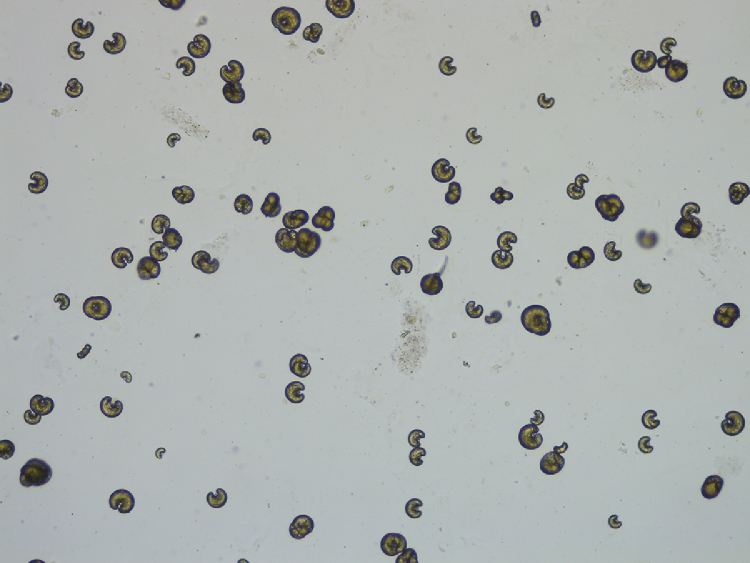
Xanthine crystals from a ferret treated with allopurinol. Unstained sediment (x40 objective).

In the first report on leishmaniosis in naturally infected domestic ferret an empirically based protocol with allopurinol plus meglumine antimoniate was established (Giner et al., 2020b). Meglumine antimoniate was prolonged during 8 weeks due to the fact that the parasite culture was still positive and allopurinol was administered *sine die*. After finishing meglumine antimoniate administration, the domestic ferret was classified as apparently healthy. Six months since treatment was initiated, xanthinuria was detected which was related to the allopurinol treatment (Fig. 5).

In the second domestic ferret leishmaniosis case report published, an anti-*Leishmania* therapeutic protocol was established with miltefosine during 28 days and allopurinol *sine die* (Giner et al., 2021a). In contrast, in this second case report, the presence of xanthinuria associated with allopurinol treatment was not observed in urine sediment during the follow-up. This urinary adverse effect of allopurinol should be considered as dependent of each treated animal (Giner et al., 2020b). In the other mustelid with clinical leishmaniosis, allopurinol alone was administered during 3 months and the clinical signs disappeared.

Miltefosine interferes in parasite metabolic pathways and the induction of apoptosis (Soto & Soto, 2006). It is considered the second-choice drug used for leishmaniosis in dogs (Dias et al., 2020; Solano-Gallego et al., 2011). The main side effects in dogs are usually vomiting and diarrhea (Solano-Gallego et al., 2011).

The most commonly used treatments for CanL are two different protocols including meglumine antimoniate plus allopurinol protocol, or miltefosine plus allopurinol protocol. Dogs treated with meglumine antimoniate plus allopurinol protocol have a lower risk of relapse compared to dogs treated with miltefosine plus allopurinol protocol (Manna et al., 2014). In FeL, treatment information is very limited based on empirically based treatment protocols. Allopurinol alone and meglumine antimoniate alone are the most common drugs used (Pennisi et al., 2015; Pereira & Maia, 2021).

### 3.6 Prevention

Although future research is needed to analyze the role of domestic ferrets as hosts for *L. infantum*, it is necessary to develop preventive measures against the parasite in this species to contribute to the decline in the infection prevalence in endemic areas and to reduce the *Leishmania*´s impact of developing a clinical disease in these animals.

Current preventative measures in dogs are based on vaccines against *Leishmania,* the administration of domperidone as immune-modulator agent (Travi & Miró, 2018) and the use of registered products with a repellent activity against sand flies (Solano-Gallego et al., 2011). In domestic ferrets, the use of these products is off-label and the application of concentrated pyrethrin and pyrethroids spot-on products labelled for dogs may cause tremors or seizures in that species (Dunayer, 2008). However, an imidacloprid 10%/permethrin 50% solution was tested in a farmed mink (*Neovison vison*) flea control study (Larsen, Siggurdsson, & Mencke, 2005) which was found to be effective without toxicity effects. Minks are a close relative species of domestic ferrets; therefore these results could be extrapolated and this solution could be used in this species. In cats, flumethrin, a synthetic piretroid with a repellent activity against sand flies can be safely used (Brianti et al., 2017). Nevertheless, further studies are required to investigate these agents as repellent effect against sand flies in domestic ferrets.

**Fig. 6:**
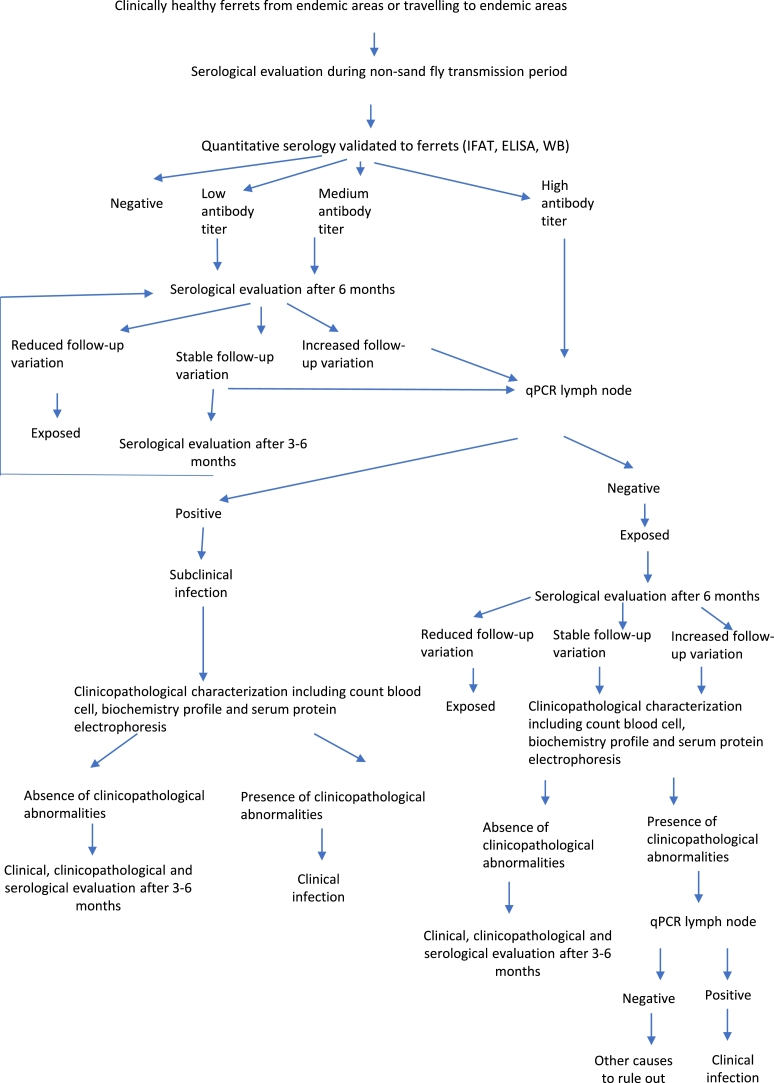
Diagnostic algorithm for clinically healthy domestic ferrets.

**Fig. 7:**
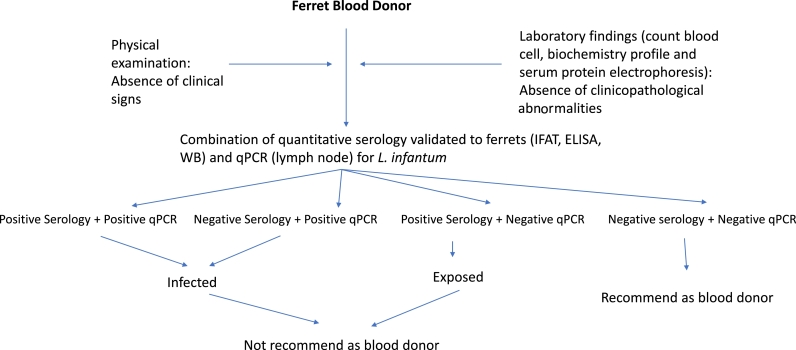
Diagnostic algorithm for clinically healthy domestic ferrets used as blood donors.

Serological screening for early detection of *Leishmania* antibodies as serologic marker of infection during non-transmission sand fly period to monitor the serological status is another preventative measure that can be implemented every year (Fig. 6). As in dogs and cats, transfusion transmitted infection associated to *Leishmania* should be considered, being necessary to test blood donors by serology and blood qPCR (Fig. 7). Another preventative measure to avoid sand fly bites could be to keep domestic ferrets indoors because sand flies are more active in the evening.

## 7 Conclusions

Leishmaniosis caused by *L. infantum* is a chronic infection affecting mainly dogs, cats, lagomorphs (Tsakmakidis et al., 2019) rodents (Alcover et al., 2021b) and other mustelids including minks (Giner et al., 2021b) and domestic ferrets could be infected. This short review highlights the need for xenodiagnosis to evaluate the infectiousness of domestic ferrets to sand flies and the possibility that infected domestic ferrets may represent an additional domestic reservoir for the parasite impelling the study and detection of clinically affected and subclinically seropositive domestic ferrets. Some further studies to adaptation and validation of serological techniques for ferrets are necessary, as serology is still the main tool of the monitoring and control the *L. infantum* infection in all animal species. In this sense, research should be carried out to expand the knowledge about *L. infantum* in mustelids.

## Funding information

This research did not receive any specific grant from funding agencies in the public, commercial, or not-for-profit sectors.
